# The Differential Relation of Emotional, Physical, and Sexual Abuse Histories to Antidepressant Treatment Remission and Persistence of Anhedonia in Major Depression: A CAN-BIND-1 Report

**DOI:** 10.1177/07067437231156255

**Published:** 2023-02-13

**Authors:** Kate L. Harkness, Trisha Chakrabarty, Sakina J. Rizvi, Raegan Mazurka, Lena Quilty, Rudolf Uher, Roumen V. Milev, Benicio N. Frey, Sagar V. Parikh, Jane A. Foster, Susan Rotzinger, Sidney H. Kennedy, Raymond W. Lam

**Affiliations:** 1 Department of Psychology, 4257 Queen's University, Kingston, ON, Canada; 2 Department of Psychiatry, 8166 University of British Columbia, Vancouver, BC, Canada; 3 Centre for Depression and Suicide Studies, 10071 St. Michael's Hospital, Toronto ON, Canada; 4 Department of Psychiatry, 7938 University of Toronto, Toronto, ON, Canada; 5 Department of Psychiatry, 3688 Dalhousie University, Halifax, NS, Canada; 6 Centre for Addiction and Mental Health, 7938 University of Toronto, Toronto, ON, Canada; 7 Department of Psychiatry, 4257 Queen's University, and Providence Care, Kingston, ON, Canada; 8 Department of Psychiatry and Behavioural Neurosciences, 3710 McMaster University, Hamilton, ON, Canada; 9 Mood Disorders Program, St. Joseph's Healthcare Hamilton, Hamilton, ON, Canada; 10 Department of Psychiatry, 1259 University of Michigan, Ann Arbor, MI, USA

**Keywords:** major depression, childhood maltreatment, anhedonia, antidepressant treatment

## Abstract

**Objective:**

Childhood maltreatment is a potent enviromarker of risk for poor response to antidepressant medication (ADM). However, childhood maltreatment is a heterogeneous construct that includes distinct exposures that have distinct neurobiological and psychological correlates. The purpose of the current study is to examine the differential associations of emotional, physical, and sexual maltreatment to ADM outcome and to examine the unique role of anhedonia in driving poor response in patients with specific maltreatment histories.

**Methods:**

In a multicentre clinical trial of major depression, 164 individuals were assessed for childhood emotional, physical, and sexual maltreatment with a contextual interview with independent, standardized ratings. All individuals received 8 weeks of escitalopram, with nonresponders subsequently also receiving augmentation with aripiprazole, with outcomes measured with depression rating scales and an anhedonia scale.

**Results:**

Greater severity of emotional maltreatment perpetrated by the mother was a significant and direct predictor of lower odds of week 16 remission (odds ratio [OR] = 1.68, *P* = 0.02). In contrast, the relations of paternal-perpetrated emotional maltreatment and physical maltreatment to week 16 remission were indirect, mediated through greater severity of anhedonia at week 8.

**Conclusions:**

We identify emotional maltreatment as a specific early exposure that places patients at the greatest risk for nonremission following pharmacological treatment. Further, we suggest that anhedonia is a key symptom domain driving nonremission in patients with particular maltreatment histories.

## Résumé

**Objectif:**

Les mauvais traitements dans l’enfance sont un puissant marqueur environnemental du risque d’une mauvaise réponse aux médicaments antidépresseurs (MAD). Toutefois, les mauvais traitements dans l’enfance sont une construction hétérogène qui inclut des expositions distinctes ayant des corrélats neurobiologiques et psychologiques distincts. Le but de la présente étude est d’examiner les associations différentielles de mauvais traitements émotionnels, physiques et sexuels avec les résultats des MAD et d’examiner le rôle unique de l’anhédonie qui entraîne une mauvaise réponse chez les patients qui ont des antécédents spécifiques de mauvais traitements.

**Méthodes:**

Dans un essai clinique multicentrique de la dépression majeure, 164 personnes ont été évaluées relativement aux mauvais traitements émotionnels, physiques et sexuels dans l’enfance et ont répondu à une entrevue contextuelle avec des notations indépendantes, standardisées. Toutes les personnes ont reçu 8 semaines d’escitalopram, et les non-répondants ont reçu subséquemment également une augmentation d’aripiprazole, et les résultats ont été mesurés avec des échelles d’évaluation de la dépression et une échelle d’anhédonie.

**Résultats:**

Les mauvais traitements émotionnels plus graves perpétrés par la mère étaient un prédicteur significatif et direct de probabilités plus faibles d’une rémission à 16 semaines (RC = 1,68, *P* = 0,02). En revanche, les relations des mauvais traitements émotionnels et physiques perpétrés par le père à la rémission de 16 semaines étaient indirectes, conditionnées par la plus grande gravité de l’anhédonie à 8 semaines.

**Conclusions:**

Nous identifions les mauvais traitements émotionnels comme étant une exposition précoce spécifique qui place les patients à risque accru de non-rémission par suite d’un traitement pharmacologique. En outre, nous suggérons que l’anhédonie est un domaine de symptômes principal entraînant la non-rémission chez les patients ayant des antécédents particuliers de mauvais traitements.

## Introduction

Major depressive disorder (MDD) affects close to 300 million people globally and is the leading worldwide cause of disability.^1^ A key contributor to this burden is the disorder's persistent course, even following multiple treatment attempts. Notably, in trials of antidepressant medication (ADM), sustained remission rates average only 40% to 50%.^2^ Identifying predictors of treatment response, and particularly markers that are easy to assess in the clinic, is a crucial step toward developing more personalized and effective treatments.^3^

Childhood maltreatment, including a history of emotional, physical, and/or sexual abuse, represents a particularly promising marker. Childhood maltreatment is the strongest developmental risk factor for MDD,^4^ and among depressed individuals, those with a history of childhood maltreatment have a more severe and persistent illness than those without.^5^ Meta-analytic data indicate an overall significant odds ratio (OR) of 1.43 for the relation of childhood maltreatment to poor response across trials of ADM, psychotherapy, and their combination.^5^ However, studies published since this meta-analysis reveal an inconsistent relation between childhood maltreatment and ADM outcome.^6,7^ For example, in the International Study to Predict Optimized Treatment for Depression trial (iSPOT-d),^8^ childhood maltreatment more strongly predicted poor response in patients randomized to sertraline than in those randomized to escitalopram (ESC) or venlafaxine. An additional trial reported that maltreatment predicted poor response, but only in patients receiving ADMs that had a weak affinity for the serotonin transporter (e.g., tricyclic ADMs or serotonin-norepinephrine reuptake inhibitors).^9^

The real promise of personalized medicine requires consideration of the prognostic significance of individual differences at the level of the risk marker (maltreatment) and the syndrome. Therefore, part of the reason for discrepancies in findings across studies may be due to the failure to consider the heterogeneous nature of childhood maltreatment and MDD.

First, childhood maltreatment is a broad construct that includes distinct exposures that are associated with distinct neurobiological correlates^10^ and prognostic impacts.^11^ Preliminary evidence from cross-sectional studies suggests that emotional maltreatment, particularly perpetrated by the mother, is more strongly associated with depression onset and severity than physical or sexual maltreatment.^11,12^ In contrast, maltreatment from the father appears to predict other outcomes, such as anxiety psychopathology and revictimization.^11,13^ Second, the relation of childhood maltreatment to ADM outcome may be driven by individual differences in specific domains within the broad MDD syndrome. Anhedonia—the inability to experience pleasure or enjoyment—is a strong candidate domain. Childhood maltreatment robustly predicts symptoms of anhedonia and blunted behavioural and neurobiological indicators of reward sensitivity in both healthy individuals and patients with MDD.^14,15^ Further, anhedonia predicts unfavourable treatment outcomes in MDD and is less responsive to selective serotonin reuptake inhibitor (SSRI) treatment than other symptom domains.^16,17^ This raises the possibility of anhedonia as an endophenotype that both emerges from maltreatment history and portends a negative course in ADM treatment.

To our knowledge, no studies have examined whether treatment efficacy in depression is differentially impacted by emotional versus physical versus sexual maltreatment. Further, no studies to our knowledge have examined change in anhedonia as a differential mediator of ADM outcome in those with histories of emotional versus physical versus sexual maltreatment. These are important questions as identifying which specific childhood histories are the strongest predictors of nonresponse may identify patients to be targeted for more rigorous intervention. Further, results bearing on mechanisms have the potential to inform the development of novel treatments targeting the domains driving nonremission.

We examine our research questions in the context of the 16-week, 6-site Canadian Biomarker Integration Network in Depression (CAN-BIND)-1 trial, in which patients with MDD were treated with ESC for 8 weeks; nonresponders were then augmented with aripiprazole (ARI)—a partial D2 receptor agonist that elevates dopaminergic signalling—for an additional 8 weeks.^18,19^ ARI has been shown to specifically reverse motivational anhedonia in rat models^20^ and to increase reward sensitivity (behavioural activation) in humans.^21^

We hypothesized that (1) emotional maltreatment, particularly perpetrated by the mother, will emerge as the strongest negative predictor of remission status at week 16 relative to physical or sexual maltreatment; (2) greater severity of emotional maltreatment, but not physical or sexual maltreatment, will be significantly associated with greater odds of requiring ARI augmentation (i.e., treatment nonresponse at week 8); and (3) severity of anhedonia at week 8 will significantly mediate the relation of emotional maltreatment to week 16 remission status.

## Methods

### Participants

The current study involved a secondary analysis of 164 outpatients in a current episode of MDD who completed assessments of childhood maltreatment and anhedonia during the 6-site CAN-BIND-1 trial (ClinicalTrials.gov identifier: NCT01655706).^3,18,19,22^ All participants provided written, informed consent, and all procedures were approved by each center's institutional research ethics boards and complied with ethical standards of the relevant national and institutional committees on human experimentation and with the Helsinki Declaration of 1975, as revised in 2008.

Inclusion criteria were as follows: (1) 18–60 years old, (2) Diagnostic and Statistical Manual of Mental Disorders (DSM-IV-TR) criteria for a current episode of MDD as assessed with the Mini-International Neuropsychiatric Interview,^23^ (3) current episode duration of at least 3 months, (4) free of psychotropic medications for >5 half-lives of recent antidepressant, (5) Montgomery-Åsberg Depression Rating Scale (MADRS)^24^ score ≥24, and (6) fluent in English. Exclusion criteria were: (1) bipolar disorder, psychosis, current substance abuse, (2) acute suicidality, (3) neurological disorder/head trauma/unstable medical condition, (4) pregnant/breastfeeding, (5) nonresponse to >4 adequate pharmacologic trials, (6) previous failure or intolerance to ESC or ARI, and (7) psychotherapy initiated in the past 3 months.

A total of 211 patients were entered into the trial and completed baseline measures.^3,17^ Of these, 28 did not complete the childhood maltreatment interview. A further 19 did not complete the full 16-week trial, leaving a final sample for the current analyses of 164. The included patients did not differ from those excluded on any demographic, clinical, or maltreatment variable (all *P*s>0.05). GPower^25^ was used to estimate the sample size required to detect a small effect in a logistic regression model, assuming moderate correlations among predictors. Setting alpha at 0.05 and power at 0.90 yielded an OR of 1.86 and a sample size of 148.

### Study Procedure and Measures

All patients started open-label ESC at 10 mg daily, increasing to 20 mg daily at week 2 or 4 based on clinician judgement.^3,18^ Response at week 8 was defined as ≥50% MADRS reduction from baseline. Responders remained at the effective dose of ESC for an additional 8 weeks. Nonresponders received ARI augmentation for 8 weeks, flexibly dosed between 2 and 10 mg daily as tolerated. The primary dependent variable in the current report was remission status at week 16. Remission was defined as a MADRS score of <10 at week 16. We chose remission as our clinical outcome instead of response because requiring a final MADRS <10 ensures that the outcome is below clinical thresholds for MDD.^26^

**Childhood Maltreatment.** The Childhood Experience of Care and Abuse (CECA) is a semistructured, contextual interview assessing exposure to emotional, physical, and sexual maltreatment prior to age 18.^27^ Interviews were conducted via secure videoconference at week 4 by doctoral-level clinicians who were trained and supervised throughout the trial by the primary author. Maltreatment subscales were subsequently rated for severity by independent judges on a scale from 1—*little/none* to 4—*marked* based on contextual features (e.g., frequency, chronicity, and degree of injury). A manual containing rating rules and hundreds of anchored exemplars was used for standardization.^27^ Subscales included the following: (1) emotional maltreatment: hostility and criticism directed toward the child by parents; (2) physical maltreatment: violence toward the child by parents; and (3) sexual maltreatment: age-inappropriate and/or nonconsensual sexual activity directed toward the child by any perpetrator. For emotional and physical maltreatment, separate ratings were provided for each parent, and the highest of the parent ratings was used in analyses. For sexual maltreatment, separate ratings were provided for each perpetrator, and the highest rating was used in analyses.

**Anhedonia.** The Dimensional Anhedonia Rating Scale (DARS)^28^ is a 17-item questionnaire assessing desire, motivation, and consummatory pleasure across 4 domains (hobbies/activities, food/drink, social activities, and sensory experience). Higher scores represent greater interest or pleasure. DARS scores from the baseline and week 8 visits were used in analyses.

### Data Analysis

SPSS statistical software version 26.0 was used for all analyses. Preliminary univariate analyses identified potential demographic or clinical covariates to include in our final models. Our first research question (maltreatment predicts remission status) was tested with a logistic regression model. The dependent variable was week 16 remission status. The predictors included treatment arm (ESC vs. ESC + ARI), and physical, sexual, and emotional maltreatment scores, entered simultaneously. The resulting parameters, thus, provide an estimate of the statistical relation of the specific maltreatment type, over and above the variance accounted for by the other maltreatment types. Receiver Operating Curves (ROCs) were fit to determine which predictors maximized the sensitivity and specificity of prediction. Further, for each type of maltreatment that emerged as a significant predictor of remission, we examined in a follow-up logistic regression model whether treatment arm significantly moderated the relation of maltreatment to week 16 remission status by including the interaction term of treatment arm with the relevant childhood maltreatment variable.

Our second hypothesis was tested with a logistic regression model predicting treatment arm (i.e., week 8 response) from physical, sexual, and emotional maltreatment scores, entered simultaneously. Our third hypothesis (maltreatment predicts week 16 remission through maintenance of anhedonia) was tested with mediation models conducted in PROCESS (Model 4).^29^ Separate models were specified for each type of maltreatment (independent variable: emotional vs. physical vs. sexual). A standardized residual of Week 8 DARS score regressed on baseline DARS score (i.e., change in anhedonia over the first 8 weeks of treatment) was the mediator, and week 16 remission status was the dependent variable. For each model, we used 5,000 bootstrap estimates to obtain 95% bias-corrected confidence intervals (CIs) for the conditional indirect effects. If the CIs for the indirect effect did not overlap with zero, we concluded that the indirect effect was statistically significant.^29^ A significant direct effect is not a necessary component of mediation models as mediation hypotheses refer solely to the indirect effect.^29–31^ Therefore, we proceeded to test mediation even when the direct paths were not significant.

## Results

Remitters and nonremitters at week 16 did not differ significantly in sex, age, ethnicity, years of education, income, number of previous MDD episodes, duration of illness, age at first onset, or the presence of a comorbid disorder (see Table 1). Further, all of the models below were robust to inclusion of these covariates. As expected, nonremitters had significantly higher symptom scores at all time points, and were significantly more likely to have required ARI augmentation, than remitters.

**Table 1.** Demographic and Clinical Characteristics of the Completer Sample Stratified by Week 16 Remission Status (*n* = 164).

Characteristic	Remitters (*n* = 98)		Nonremitters (*n* = 66)		Statistic
	Mean	SD	Mean	SD	*t*
Age (years)	35.23	12.24	37.05	13.42	0.89
Years of education	14.17	2.00	14.11	2.06	0.19
	*n*	%	*n*	%	χ^2^
Female	62	63	38	58	0.54
Ethnicity					6.15
White	75	77	43	65	
Black	3	3	4	6	
Asian	9	9	14	21	
Other	11	11	5	8	
Household income^1^					2.48
$10,000–$24,999	17	20	18	31	
$25,000–$74,999	42	49	23	39	
$75,000–$149,000	21	25	14	24	
≥$150,000	5	6	4	6	
	Mean	*SD*	Mean	*SD*	*t*
Baseline MADRS	28.92	5.12	31.21	5.79	2.67*
Week 16 MADRS	4.73	3.16	18.76	6.92	17.55**
Baseline DARS	36.24	13.57	30.00	14.58	2.81*
Week 8 DARS	46.09	13.16	32.30	15.56	6.11*
Number of past episodes	3.18	2.88	3.06	3.74	0.24
Age at first MDD onset	20.53	8.38	21.47	12.14	0.58
Duration of illness (years)	14.72	12.04	15.92	12.73	0.60
	*n*	%	*n*	%	χ^2^
Comorbid diagnosis	35	36	31	47	2.08
Treatment arm					23.55**
ESC	60	61	15	23	
ESC + ARI	38	39	51	77	

^1^ 20 patients did not disclose their income, leaving *n* = 144 for this analysis. *P < 0.01; **P < 0.001.

*Note*: MADRS = Montgomery-Asberg Depression Rating Scale; DARS = Dimensional Anhedonia Rating Scale; ESC = escitalopram; ARI = aripiprazole; MDD = major depressive disorder.

### Childhood Maltreatment and Week 16 Remission

Intercorrelations among the maltreatment variables, and estimated marginal means stratified by week 16 remission status and treatment arm (ESC vs. ESC + ARI), are provided in Table 2. Despite significant intercorrelation among the maltreatment variables, none of the variance inflation factor (VIF) values exceeded 4, suggesting that our models did not have a problem with multicollinearity.^32^

**Table 2.** Intercorrelations Among Maltreatment Types and Descriptive Statistics Stratified by Remission Status and Treatment Arm.

	Week 16 remission				Treatment arm (week 8 response)								
	Yes (*N* = 98)		No (*N* = 66)		ESC (*N* = 74)		ESC + ARI (*N* = 88)						
	Mean	SE	Mean	SE	Mean	SE	Mean	SE	VIF	1	2	3	4
1. Emotional maltreatment	1.99	0.09	2.33	0.11	2.06	0.10	2.19	0.09	1.52	1.00			
2. Physical maltreatment	2.22	0.11	1.97	0.13	2.15	0.12	2.09	0.11	1.57	.54*	1.00		
3. Sexual maltreatment^2^	1.90	0.13	1.57	0.16	1.81	0.14	1.73	0.13	1.24	.37*	.41*	1.00	
4. Maternal EM	1.50	0.09	1.98	0.11	1.68	0.10	1.75	0.09	1.02	.77*	.47*	.40*	1.00
5. Paternal EM^1^	1.74	0.09	1.71	0.11	1.62	0.10	1.82	0.09	1.03	.65*	.33*	.14	.13

^1^ Nine patients did not have a father figure (*n* = 155); ^2^two patients did not disclose their sexual maltreatment history (*n* = 162); *P < 0.001.

*Note*: Emotional maltreatment, physical maltreatment, and sexual maltreatment were entered in the primary model; maternal EM and paternal EM were entered in a separate secondary model. Means are estimated marginal means controlling for treatment arm (for week 16 remission means only) and the other types of maltreatment; EM = emotional maltreatment; SE = standard error; VIF = variance inflation factor.

Parameter estimates for the multivariate logistic regression model predicting week 16 remission status are provided in Table 3. As hypothesized, over and above the association of treatment arm to remission status, greater severity of emotional maltreatment significantly predicted a lower odds of remission at week 16, whereas the relations of physical and sexual maltreatment to remission status were not significant. The OR indicates that each point increase in emotional maltreatment severity was associated with a 68% greater odds of being in the nonremitter group. ROC analysis indicated that areas under the curve (AUC) were significant for the full model (AUC = 0.76, standard error [SE] = 0.038, *P* < 0.001 [95% CI_,_ 0.69 to 0.84]) and the model including just the maltreatment variables (AUC = 0.64, SE = 0.044, *P* = 0.003 [95% CI_,_ 0.55 to 0.73]). However, AUC for the model further excluding emotional maltreatment did not even approach significance (AUC = 0.52, SE = 0.046, *P* = 0.72 [95% CI_,_ 0.43 to 0.60]). That is, the logistic regression classified remission group significantly better than by chance only when emotional maltreatment and/or treatment arm were in the model. Follow-up analysis indicated that the interaction of treatment arm and emotional maltreatment on remission status was not significant (OR = 0.84, *P* = 0.63, 95% CI, 0.41 to 1.73).

**Table 3.** Parameter Estimates for Logistic Regression Model Predicting Week 16 Remission.

	B	SE	Wald	*P*	OR	95% CI (OR)
Treatment arm	1.79	0.38	22.51	<0.001	6.00	2.86 to 12.57
Emotional maltreatment	0.52	0.22	5.41	0.02	1.68	1.08 to 2.61
Physical maltreatment	−0.27	0.19	2.07	0.15	0.76	0.53 to 1.10
Sexual maltreatment	−0.22	0.15	2.04	0.15	0.80	0.59 to 1.09

*Note*. SE = standard error; OR = odds ratio; 95% CI (OR) = 95% confidence interval around the OR.

A secondary logistic regression model revealed that over and above treatment arm (OR = 6.10, *P* < 0.001, 95% CI_,_ 2.86 to 13.02), higher severity of emotional maltreatment perpetrated by the *mother*, OR = 1.53, *P* = 0.03, 95% CI, 1.05 to 2.22, significantly predicted lower odds of remission, whereas emotional maltreatment perpetrated by the father did not, OR = 0.83, *P* = 0.40, 95% CI, 0.54 to 1.28. Each point increase in severity of maternal emotional maltreatment was associated with 53% greater odds of being in the nonremitter group. As above, a follow-up analysis revealed no evidence of a significant interaction between maternal emotional maltreatment and treatment arm, OR = 0.79, *P* = 0.54, 95% CI, 0.36 to 1.70.

### Childhood Maltreatment and Week 8 Response

A multivariate logistic regression model predicting need for ARI augmentation at week 8 (i.e., week 8 response) from severity of emotional, physical, and sexual maltreatment, entered as a block, was not significant, χ^2^(3) = 1.19, *P* = 0.76, and none of the maltreatment types was associated with a significant parameter estimate, ORs = 0.68–1.23, all *P*s > 0.29 (see Table 2 for marginal means).

### Childhood Maltreatment, Week 8 Anhedonia, and Week 16 Remission

**Figure 1.**
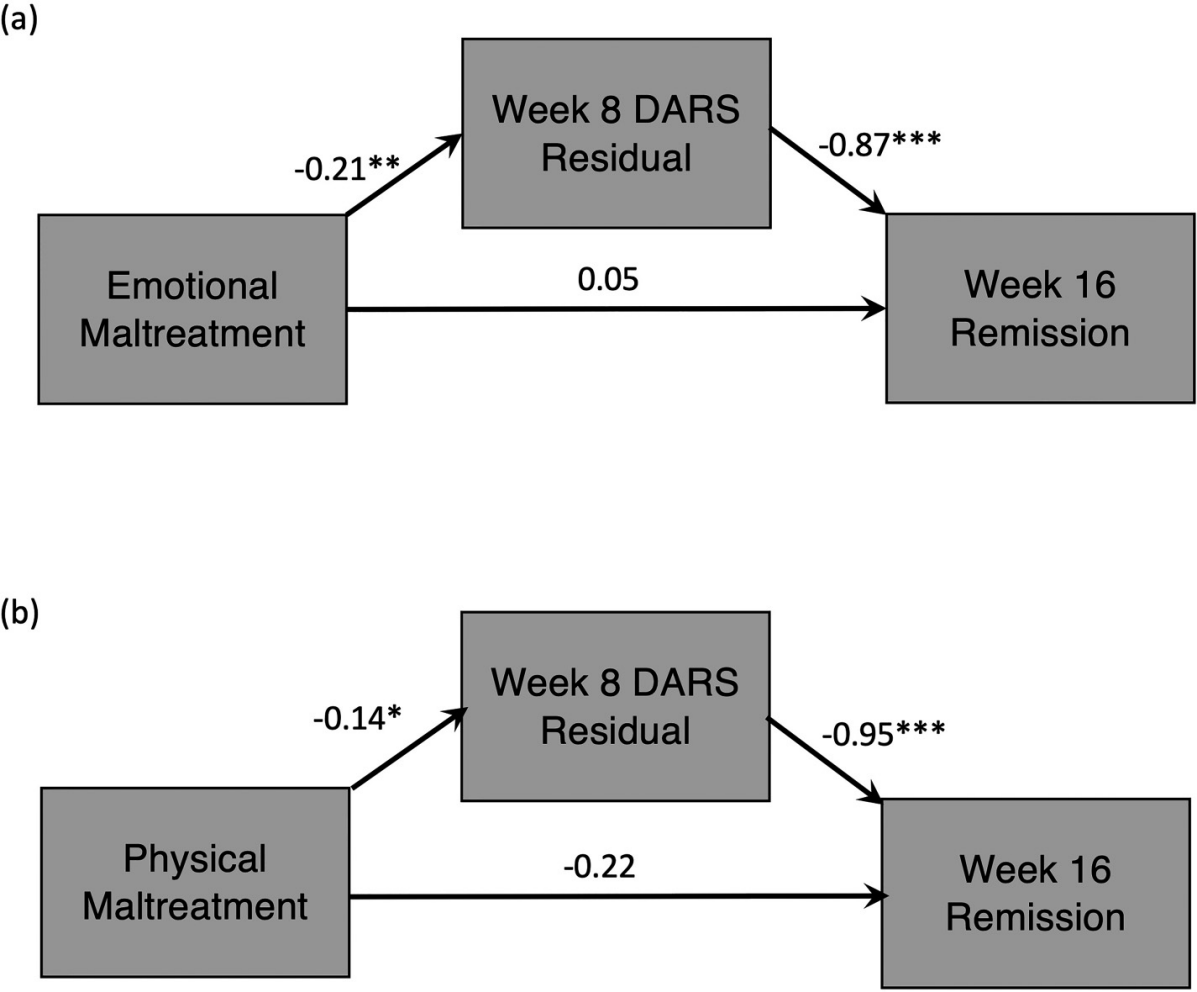
Greater severity of (a) emotional maltreatment and (b) physical maltreatment predicted less change in anhedonia from baseline to week 8, which subsequently significantly predicted a greater likelihood of nonremission to escitalopram or escitalopram + aripiprazole at week 16. Parameter estimates for indirect effects are provided in the text. *P < 0.05; **P < 0.01.

However, lower scores on the week 8 DARS residual (i.e., less change from baseline, and thus, greater severity of week 8 anhedonia) were significantly correlated with greater severity of emotional maltreatment, *r* = −0.21, *P* = 0.008, and physical maltreatment, *r* = −0.18, *P* = 0.02. Therefore, these two maltreatment variables met the criteria for mediation. Coefficients for the direct paths are presented in Figure 1. In both models, higher severity of maltreatment was significantly predictive of lower week 8 DARS residual (less change in anhedonia), and lower week 8 DARS residual significantly predicted greater odds of being in the nonremitter group at week 16. The indirect relation of maltreatment to remission status through a change in anhedonia was significant in the model containing emotional maltreatment, *B* = 0.06, SE = 0.10, 95% CI, 0.03 to 0.41, and that containing physical maltreatment, *B* = 0.31, SE = 0.07, 95% CI, 0.02 to 0.29.

Follow-up mediation models revealed that the indirect effect on week 16 remission status through a change in anhedonia was *not* significant for emotional maltreatment perpetrated by the mother, *B* = 0.15, SE = 0.09, 95% CI, −0.004 to 0.36, but was significant for emotional maltreatment perpetrated by the father, *B* = 0.20, SE = 0.11, 95% CI, 0.43 to 0.008.

An open question raised by the models above is to what extent the mediation effects are *specific* to symptoms of anhedonia versus overall severity of depression symptoms. It was not appropriate to include a change in MADRS score (week 8 MADRS residual) as a covariate in the above models because the baseline MADRS score is central to the definition of the outcome variable, and, as expected, the MADRS and DARS residual scores were very highly correlated, *r* = −0.68, *P* < 0.001 (as were the raw baseline and week 8 scores). Thus, inclusion of MADRS residual scores as a covariate would have resulted in a confounded and oversaturated model. However, the direct zero-order associations of emotional, physical, and sexual maltreatment to change in MADRS from baseline to week 8 did not even approach significance (*r* = 0.08, *P* = 0.31; *r* = −0.009, *P* = 0.91; and *r* = 0.07, *P* = 0.40, respectively). Therefore, the change in MADRS score did not meet the criteria as a mediator, indicating that the above mediation effects are specific to anhedonia and do not extend to overall depression severity.

## Discussion

In a large multicentre trial that included rigorous contextual assessment of childhood maltreatment history, we found that greater severity of mother-perpetrated emotional maltreatment directly predicted lower odds of remission following 16 weeks of treatment with ESC or ESC + ARI. Further, emotional maltreatment perpetrated by the father and physical maltreatment were indirectly associated with remission status through greater severity of (less change in) anhedonia from baseline to week 8. The current design provided a particularly conservative test of our research question. The effect of emotional maltreatment was robust when controlling for overlapping variance with physical and sexual maltreatment, and ROC analysis indicated that a model containing only physical and sexual maltreatment did not predict remission better than chance. Further, the effect of emotional maltreatment on remission at week 16 was robust even over and above the very strong association of response at week 8. Previous cross-sectional research has found that emotional maltreatment is also significantly more strongly associated than other forms of maltreatment with the onset and severity of MDD.^11,12^ Thus, emotional maltreatment may represent an especially strong enviromarker that could be used in clinical practice to identify patients who require more rigorous intervention.

Higher severity of emotional and physical maltreatment was associated with greater severity of (less change in) anhedonia following 8 weeks of ESC treatment. Further, the relations of emotional maltreatment perpetrated by the father and physical maltreatment to remission status were significantly *and specifically* mediated through anhedonia. That is, it was the specific domain of anhedonia, and not depression severity generally, that significantly mediated the relation between these distinct exposures and remission. None of the maltreatment types were significantly associated with a change in the overall severity of depression symptoms from baseline to week 8. Therefore, our results suggest that childhood maltreatment may play a role in driving resistance specifically of anhedonia symptoms to SSRI treatment. And, by rigorously assessing heterogeneous maltreatment exposures, we provide novel evidence that these effects are *specific* to paternal emotional maltreatment and physical maltreatment. This is important and echoes calls in the literature to acknowledge that the very broad maltreatment construct is made up of distinct exposures that have distinct neurobiological, affective, and cognitive correlates.^33^

Symptoms of anhedonia are more strongly related to threat neurocircuitry than are symptoms of general distress in depression.^34^ Therefore, maltreatment exposures that are experienced as particularly threatening may be especially likely to affect reward functioning.^10,35^ Physical maltreatment is, by definition, an exposure that involves direct threat, with marked exposures involving repeated beating, often around the head or with an implement. There is also evidence that emotional maltreatment from the father is experienced as more directly threatening than that perpetrated by the mother.^36^ In the current qualitative CECA interview transcripts, participants reported that fathers were more often hostile and threatening in their tone, and participants were more likely to use words such as “angry” and “mean,” and to report being “frightened,” when describing emotional maltreatment perpetrated by fathers relative to mothers. More targeted research with individuals recruited specifically on the basis of their maltreatment histories is required to more deeply explore the differential associations reported here.

Maltreatment predicted remission over and above treatment arm (ESC vs. ESC + ARI). Therefore, childhood maltreatment may represent a marker of a more treatment-refractory set of patients who warrant multilevel investigation. In particular, future research is needed to examine other treatment strategies (e.g., new pharmacotherapy augmentation strategies^37,38^ and/or cognitive or behavioural activation therapy^39–41^) that may show greater efficacy in treating individuals with this severe history.

Limitations include the retrospective nature of the CECA, which raises concerns about depressive recall bias. However, contextual interview measures of childhood maltreatment are less subject to recall bias than traditional self-report measures because ratings are independent and standardized to anchored exemplars.^42^ The CECA, in particular, is considered the “gold standard.”^43^ Meta-analysis has also indicated that maltreatment retrospectively assessed using contextual interview measures is more strongly associated with reports of maltreatment taken at the time of the abuse than is maltreatment retrospectively assessed by self-report checklist.^44^ Relatedly, a smaller percentage of the sample endorsed sexual maltreatment (40%) than physical (55%) or emotional (67%) maltreatment, which may have limited our ability to detect effects for sexual maltreatment.

Further, the MADRS includes 1 item related to anhedonia (item 8 “Inability to feel”), raising the potential that our mediator may be confounded with our outcome. However, our measure of anhedonia assesses the much broader domain, capturing interest, motivation, effort, and enjoyment of rewards across several areas of life. Nevertheless, conclusions would be strengthened through future research including behavioural tasks or neurophysiological markers of anhedonia. Finally, it was beyond our scope to examine the developmental timing of maltreatment, proximal stressful life events, or additional psychobiological factors that could potentially mediate or moderate the relation between childhood maltreatment and treatment response.

Childhood maltreatment is widely regarded as one of the strongest risk factors for the onset and treatment resistance of MDD. The current results identify which specific early exposures place patients at the greatest risk, thereby allowing these patients to be assigned for more rigorous intervention. Further, they have the potential to inform the development of novel treatments targeting the domains driving nonremission, specifically anhedonia, with the ultimate goal of reducing the burden associated with MDD.

## References

[1] World Health Organization. Depression and other common mental disorders: Global health estimates. January 3, 2017. https://www.who.int/publications/i/item/depression-global-health-estimates.

[2] Trivedi MH Daly EJ . Treatment strategies to improve and sustain remission in major depressive disorder. Dialogues Clin Neurosci. 2008;10:377-384. doi: 10.31887/DCNS.2008.10.4/mhtrivedi. 19170395 PMC3181893

[3] Kennedy SH Downar J Evans KR , et al. The Canadian biomarker integration network in depression (CAN-BIND): advances in response prediction. Curr Pharm Des. 2012;18:5976-5989. doi: 10.2174/138161212803523635. 22681173

[4] Lippard ETC Nemeroff CB . The devastating clinical consequences of child abuse and neglect: increased disease vulnerability and poor treatment response in mood disorders. Am J Psychiatry. 2020;177:20-36. doi: 10.1176/appi.ajp.2019.19010020. 31537091 PMC6939135

[5] Nanni V Uher R Danese A . Childhood maltreatment predicts unfavorable course of illness and treatment outcome in depression: a meta-analysis. Am J Psychiatry. 2012;169:141-151. doi: 10.1176/appi.ajp.2011.11020335. 22420036

[6] Perna G Daccò S Alciati A , et al. Childhood maltreatment history for guiding personalized antidepressant choice in major depressive disorder: preliminary results from a systematic review. Prog Neuropsychopharmacol Biol Psychiatry. 2021;107:110208. doi: 10.1016/j.pnpbp.2020.110208. 33338557

[7] Harkness KL Bagby RM Kennedy SH . Childhood maltreatment and differential treatment response and recurrence in adult major depressive disorder. J Consult Clin Psychol. 2012;80:342-353. doi: 10.1037/a0027665. 22428942

[8] Williams LM Debattista C Duchemin AM , et al. Childhood trauma predicts antidepressant response in adults with major depression: data from the randomized international study to predict optimized treatment for depression. Transl Psychiatry. 2016;6:e799. doi: 10.1038/tp.2016.61. 27138798 PMC5070060

[9] Quilty LC Marshe V Lobo DSS , et al. Childhood abuse history in depression predicts better response to antidepressants with higher serotonin transporter affinity: a pilot investigation. Neuropsychobiology. 2016;74:78-83. doi: 10.1159/000453549. 28064281

[10] McLaughlin KA Sheridan MA . Beyond cumulative risk: a dimensional approach to childhood adversity. Curr Dir Psychol Sci. 2016;25:239-245. doi: 10.1177/0963721416655883. 27773969 PMC5070918

[11] Vallati M Cunningham S Mazurka R , et al. Childhood maltreatment and the clinical characteristics of major depressive disorder in adolescence and adulthood. J Abnorm Psychol. 2020;129:469-479. doi: 10.1037/abn0000521. 32237880

[12] Mandelli L Petrelli C Serretti A . The role of specific early trauma in adult depression: a meta-analysis of published literature. Childhood trauma and adult depression. Eur Psychiatry. 2015;30:665-680. doi: 10.1016/j.eurpsy.2015.04.007. 26078093

[13] Cunningham S Goff C Bagby RM , et al. Maternal- versus paternal-perpetrated maltreatment and risk for sexual and peer bullying revictimization in young women with depression. Child Ab Negl. 2019;89:111-121. doi: 10.1016/j.chiabu.2018.12.017. 30658172

[14] Harkness KL, Lamontagne SJ, & Cunningham S. Environmental contributions to anhedonia. In: Pizzagalli DA, & Der-Avakian A, editors. Current topics in behavioral neurosciences. Berlin, Heidelberg: Springer; 2022; 58:81-108. doi: 10.1007/7854_2021_289. 34894350

[15] Hanson JL Williams AV Bangasser DA , et al. Impact of early life stress on reward circuit function and regulation. Front Psychiatry. 2021;12:744690. doi: 10.3389/fpsyt.2021.744690. 34744836 PMC8563782

[16] McMakin DL Olino TM Porta G , et al. Anhedonia predicts poorer recovery among youth with selective serotonin reuptake inhibitor treatment-resistant depression. J Am Acad Child Adolesc Psychiatry. 2012 Apr 1;51:404-411. doi: 10.1016/j.jaac.2012.01.011. 22449646 PMC3536476

[17] Downar J Geraci J Salomons TV , et al. Anhedonia and reward-circuit connectivity distinguish nonresponders from responders to dorsomedial prefrontal repetitive transcranial magnetic stimulation in major depression. Biol Psychiatry. 2014;76:176-185. doi: 10.1016/j.biopsych.2013.10.026. 24388670

[18] Kennedy SH Lam RW Rotzinger S , et al. Symptomatic and functional outcomes and early prediction of response to escitalopram monotherapy and sequential adjunctive aripiprazole therapy in patients with major depressive disorder: a CAN-BIND-1 report. J Clin Psychiatry. 2019;80:18m12202. doi: 10.4088/JCP.18m12202. 30840787

[19] Lam RW Milev R Rotzinger S , et al. Discovering biomarkers for antidepressant response: protocol from the Canadian biomarker integration network in depression (CAN-BIND) and clinical characteristics of the first patient cohort. BMC Psychiatry. 2016;16:105. doi: 10.1186/s12888-016-0785-x. 27084692 PMC4833905

[20] Scheggi S De Montis M Gambarana C . Making sense of rodent models of anhedonia. Int J Neuropsychopharmacol. 2018;21:1049-1065. doi: 10.1093/ijnp/pyy083. 30239762 PMC6209858

[21] Allen TA Lam RW Milev R , et al. Early change in reward and punishment sensitivity as a predictor of response to antidepressant treatment for major depressive disorder: a CAN-BIND-1 report. Psychol Med. 2019;49:1629-1638. doi: 10.1017/S0033291718002441. 30220263

[22] Chakrabarty T Harkness KL McInerney SJ , et al. Childhood maltreatment and cognitive functioning in patients with major depressive disorder: a CAN-BIND-1 report. Psychol Med. 2020;50:2536-2547. doi: 10.1017/S003329171900268X. 31583989

[23] Sheehan DV Lecrubier Y Sheehan KH , et al. The Mini-International Neuropsychiatric Interview (M.I.N.I.): the development and validation of a structured diagnostic psychiatric interview for DSM-IV and ICD-10. J Clin Psychiatry. 1998;59(suppl 20):22-33. 9881538

[24] Montgomery SA Asberg M . A new depression scale designed to be sensitive to change. Br J Psychiatry. 1979;134:382-389. doi: 10.1192/bjp.134.4.382. 444788

[25] Erdfelder E, Faul F, Buchner A. GPOWER: a general power analysis program. Behav Res Methods Instrum Comput. 1996;28:1-11. doi: 10.3758/BF03203630.

[26] Hawley CJ Gale TM Sivakumaran T , Hertfordshire Neuroscience Research Group. Defining remission by cut off score on the MADRS: selecting the optimal value. J Affect Disord. 2002;72:177-184. doi: 10.1016/s0165-0327(01)00451-7. 12200208

[27] Bifulco A Brown GW Harris TO . Childhood experience of care and abuse (CECA): a retrospective interview measure. J Child Psychol Psychiatry. 1994;35:1419-1435. doi: 10.1111/j.1469-7610.1994.tb01284.x. 7868637

[28] Rizvi SJ Quilty LC Sproule BA , et al. Development and validation of the dimensional anhedonia rating scale (DARS) in a community sample and individuals with major depression. Psychiatry Res. 2015;229:109-119. doi: 10.1016/j.psychres.2015.07.062. 26250147

[29] Hayes AF . Introduction to mediation, moderation, and conditional process analysis: a regression-based approach. 2nd ed. New York: Guilford Press; 2018.

[30] Kenny DA Judd CM . Power anomalies in testing mediation. Psychol Sci. 2014;25:334-339. 10.1177/0956797613502676. 24311476

[31] O’Rourke HP, & MacKinnon DP. When the test of mediation is more powerful than the test of the total effect. Behav Res Methods. 2015;47(2):424-442. doi:10.3758/s13428-014-0481-z. 24903690 PMC4258193

[32] Hair J Ringle C Sarstedt M . PLS-SEM: indeed a silver bullet. J Mark Theory Pract. 2011;19:139-151. 10.2753/MTP1069-6679190202.

[33] Sheridan MA McLaughlin KA . Neurobiological models of the impact of adversity on education. Curr Opin Behav Sci. 2016;10:108-113. 10.1016/j.cobeha.2016.05.013. 29046891 PMC5642918

[34] Young KS Bookheimer SY Nusslock R , et al. Dysregulation of threat neurocircuitry during fear extinction: the role of anhedonia. Neuropsychopharmacol. 2021;46:1650-1657. doi: 10.1038/s41386-021-01003-8. PMC8280223 33833400

[35] Agrawal A Nelson EC Littlefield AK , et al. Cannabinoid receptor genotype moderation of the effects of childhood physical abuse on anhedonia and depression. Arch Gen Psychiatry. 2012;69:732-740. doi: 10.1001/archgenpsychiatry.2011.2273. 22393204 PMC3706194

[36] Neel R Becker DV Neuberg SL , et al. Who expressed what emotion? Men grab anger, women grab happiness. J Exp Soc Psychol. 2012;48:583-586. doi: 10.1016/j.jesp.2011.11.009. 22368303 PMC3285231

[37] Nuñez NA Joseph B Pahwa M , et al. Augmentation strategies for treatment resistant major depression: a systematic review and network meta-analysis. J Affect Disord. 2022;302:385-400. doi: 10.1016/j.jad.2021.12.134. 34986373 PMC9328668

[38] Papp M Gruca P Lasoń-Tyburkiewicz M , et al. Attenuation of anhedonia by cariprazine in the chronic mild stress model of depression. Behav Pharmacol. 2014;25:567-574. doi: 10.1097/FBP.0000000000000070. 25083572

[39] Forbes CN . New directions in behavioral activation: using findings from basic science and translational neuroscience to inform the exploration of potential mechanisms of change. Clin Psychol Rev. 2020;79:101860. doi: 10.1016/j.cpr.2020.101860. 32413734

[40] Dichter GS Felder JN Petty CP , et al. The effects of psychotherapy on neural responses to rewards in major depression. Biol Psychiatry. 2009;66:886-897. doi: 10.1016/j.biopsych.2009.06.021. 19726030 PMC3657763

[41] Walsh EC Eisenlohr-Moul TA Minkel J , et al. Pretreatment brain connectivity during positive emotion upregulation predicts decreased anhedonia following behavioral activation therapy for depression. J Affect Disord. 2019;243:188-192. doi: 10.1016/j.jad.2018.09.065. 30245249 PMC6411035

[42] Harkness KL Monroe SM . The assessment and measurement of adult life stress: basic premises, operational principles, and design requirements. J Abnorm Psychol. 2016;125:727-745. doi: 10.1037/abn0000178. 27254487

[43] Thabrew H de Sylva S Romans S . Evaluating childhood adversity. Adv Psychosom Med. 2012;32:35-57. doi:10.1159/000330002. 22056897

[44] Baldwin JR Reuben A Newbury JB Danese A . Agreement between prospective and retrospective measures of childhood maltreatment: a systematic review and meta-analysis. JAMA Psychiatry. 2019;76:584-593. doi: 10.1001/jamapsychiatry.2019.0097. 30892562 PMC6551848

